# Self-confidence and body image among adults engaged in daily bodybuilding exercise compared with non-exercising adults: a case-control study

**DOI:** 10.3389/fpubh.2026.1894880

**Published:** 2026-09-03

**Authors:** Hayder Ameer Jabor, Bahaa Mirza Skal, Hasan Naeem Kareem, Richard Mottershead

**Affiliations:** 1 Psychiatric and Mental Health Nursing Department, College of Nursing, University of AL-Qadisiyah, Al Diwaniyah, Iraq; 2 Medical Surgical Nursing Department, College of Nursing, University of AL-Qadisiyah, Al Diwaniyah, Iraq; 3 Community Health Nursing Department, College of Nursing, University of Al Qadisiyah, Al Diwaniyah, Iraq; 4 Psychiatric and Mental Health Department, College of Nursing, University of Baghdad, Baghdad, Iraq

**Keywords:** body image, bodybuilding, Iraq, physical exercise, psychological wellbeing, self-confidence

## Abstract

**Background:**

Regular bodybuilding and resistance training may positively influence psychological wellbeing, particularly self-confidence and body image perception. This study aimed to compare self-confidence and body image between adults engaged in daily bodybuilding exercise and non-exercising adults in Iraq.

**Methods:**

A case-control study was conducted in Al-Diwaniyah City, Iraq, between August 2025 and April 2026. A total of 188 participants were enrolled using a purposive sampling method, including 94 adults engaged in regular bodybuilding exercise and 94 non-exercising controls. Data were collected using a sociodemographic questionnaire, the Self-Confidence Scale, and the Body Image Scale. Data was analyzed using SPSS version 25. Statistical significance was considered at *p* < 0.05.

**Results:**

No significant sociodemographic differences were observed between the two groups (*p* > 0.05). Participants in the bodybuilding group demonstrated significantly higher total self-confidence scores compared with controls (148.37 ± 25.6 vs. 137.32 ± 26.7, *p* = 0.004). Significant differences were observed in several self-confidence domains, including social interaction, optimism, communication confidence, and appearance-related concerns (all *p* < 0.05). Body image scores were also significantly higher among bodybuilders (32.69 ± 7.6 vs. 29.70 ± 5.5, *p* = 0.002), indicating more positive body image perception and lower appearance-related distress. Logistic regression analysis showed that higher self-confidence scores were independently associated with increased likelihood of belonging to the bodybuilding group (OR = 0.988, 95% CI: 0.976–0.999, *p* = 0.040). Similarly, body image scores were significant predictors of group membership (OR = 0.937, 95% CI: 0.892–0.984, *p* = 0.009).

**Conclusion:**

Adults who regularly participated in bodybuilding had significantly higher self-confidence and a more positive body image than non-exercising adults. These findings indicate an association rather than a causal relationship, as individuals with higher self-confidence and a more positive body image may be more likely to engage in bodybuilding. Regular participation in bodybuilding was associated with higher self-confidence and more positive body image among adults in this study. However, because of the case-control design, the direction of this association cannot be determined.

## Introduction

Regular physical exercise is widely recognized as an important determinant of both physical and psychological wellbeing (1). Among the different forms of exercise, bodybuilding and resistance training have gained increasing popularity among adults because of their role in improving muscular strength, physical appearance, and overall fitness (2). In addition to their physical benefits, participation in regular bodybuilding exercise may also influence psychological constructs such as self-confidence and body image perception (3). These psychosocial outcomes are particularly important because they affect interpersonal relationships, emotional stability, quality of life, and social functioning (4).

Self-confidence refers to an individual's belief in their abilities, personal competence, and capacity to successfully cope with life challenges (5). It is considered an essential component of mental health and psychological adjustment (6). Individuals with high self-confidence tend to demonstrate greater emotional resilience, social competence, optimism, and academic or occupational achievement (7). Conversely, low self-confidence has been associated with anxiety, social withdrawal, negative self-evaluation, and depressive symptoms (8). Physical exercise, particularly resistance and bodybuilding training, has been reported to enhance self-confidence by improving physical performance, self-perception, and social interaction (9).

Body image is another important psychological construct that reflects individuals' perceptions, attitudes, and feelings toward their own physical appearance (10). Positive body image is associated with psychological wellbeing, higher self-esteem, and better social functioning, whereas negative body image is linked to body dissatisfaction, appearance-related distress, anxiety, eating disorders, and depression (11). Participation in bodybuilding exercise may positively influence body image through improvements in muscularity, physical appearance, and perceived attractiveness (12). However, some studies have also suggested that excessive concern with muscular development and physical appearance among bodybuilders may contribute to body dissatisfaction and distorted body image perceptions (13, 14).

Several previous studies have examined the relationship between physical exercise and psychological outcomes (15, 16). Evidence suggests that regular physical activity is associated with improved self-esteem, enhanced mood, reduced stress, and better body image perception (11). Resistance training programs specifically have demonstrated beneficial effects on self-perceived competence and psychological wellbeing among adults (1). Nevertheless, findings remain inconsistent across populations and cultural settings, and limited research has focused specifically on adults engaged in daily bodybuilding exercise in Middle Eastern communities, including Iraq.

In Iraq, bodybuilding and fitness activities have become increasingly common among young adults and university students, particularly in urban areas (17). Despite this growing interest, few studies have investigated the psychological effects of bodybuilding exercise on self-confidence and body image among Iraqi adults. Understanding these relationships may help healthcare professionals, psychologists, and fitness specialists identify the potential mental health benefits associated with regular bodybuilding exercise and develop interventions that promote healthy body perception and psychological wellbeing. Therefore, the present study aimed to compare self-confidence and body image among adults engaged in daily bodybuilding exercise with non-exercising adults using a case-control study design.

## Materials and methods

### Study design

A case-control study design was utilized to examine self-confidence and body image among adults engaged in daily bodybuilding exercise in comparison with non-exercising adults.

### Study setting and period

The study was conducted in Al-Diwaniyah City, located in Al-Qadisiyah Governorate, Iraq. Data were collected from five private bodybuilding and fitness centers where adults regularly participated in daily bodybuilding exercises and resistance training programs. These centers provide structured physical training environments that attract individuals interested in improving muscular strength, physical fitness, and body appearance. The control group participants were recruited from non-exercising adults in the general population, including students from Al-Qadisiyah University. The study was carried out over a period extending from 8th August 2025 to 1st April 2026.

### Study population

The study population included adults who regularly attended bodybuilding and fitness centers and participated in daily bodybuilding exercises, in addition to non-exercising adults from the general population, including students from Al-Qadisiyah University who served as the control group. Frequency matching was performed using age and selected sociodemographic characteristics. Gender was not included as a matching variable because it was not recorded during participant recruitment. Recruitment of the control group was conducted concurrently with the case group, and participant characteristics were monitored throughout the enrollment process to maintain similar distributions across the matching variables. Eligible participants for the control group were selected until comparable frequency distributions were achieved, thereby minimizing potential confounding due to differences in baseline characteristics.

### Eligibility criteria

Participants were eligible for inclusion if they were adults aged 18 years and above and willing to participate in the study by providing informed consent. The case group included individuals who had been regularly engaged in bodybuilding and resistance training exercises for at least 6 months, while the control group consisted of non-exercising adults who had not participated in any structured physical exercise program during the preceding 6 months. Participants were required to be able to understand and complete the study questionnaire. Individuals with less than 6 months of bodybuilding experience, those involved in other regular intensive sports or structured fitness activities (for the control group), participants with incomplete questionnaire responses, and those who refused to participate or withdrew consent were excluded from the study.

### Sample size and sampling technique

The study included a total sample of 188 participants, comprising 94 adults engaged in regular bodybuilding exercises (case group) and 94 non-exercising adults (control group). The sample size was determined based on feasibility and availability of eligible participants within the selected study settings during the data collection period. A non-probability purposive sampling technique was used to recruit participants for both groups. Bodybuilders were selected from five private bodybuilding and fitness centers in Al-Diwaniyah City, while control participants were recruited from non-exercising adults in the general population, including students from Al-Qadisiyah University, ensuring matching between groups based on age and sociodemographic characteristics to enhance comparability.

### Data collection

Interviews were conducted face-to-face with participants in bodybuilding centers for the case group, while control participants were recruited from the general population, including students from Al-Qadisiyah University, and completed the same instruments under similar conditions. The data collection process ensured privacy and confidentiality, and participants were given sufficient time to respond to all items.

The study questionnaire consisted of three main parts: (1) sociodemographic characteristics (age, marital status, educational level, and monthly income), (2) the Self-Confidence Scale, and (3) the Body Image Scale. The Self-Confidence Scale is a 48-item instrument measured on a five-point Likert scale ranging from not at all (1) to completely applies (5), assessing various dimensions of self-evaluation, social interaction, and personal competence (18, 19). The Self-Confidence Scale was selected because it measures perceived confidence in abilities, social interaction, communication, and competence across different situations. Although related to self-esteem, self-confidence reflects beliefs about one's capability to perform successfully, whereas self-esteem represents overall self-worth. Therefore, the term “self-confidence” was used to accurately describe the construct assessed by this instrument. The Body Image Scale was developed by Hopwood et al. (20) and is a 10-item self-report questionnaire assessing body image-related distress across emotional, cognitive, and behavioral domains. In the present study, responses were measured using a five-point Likert scale ranging from strongly agree (1) to strongly disagree (5). The Body Image Scale developed by Hopwood et al. (20) was selected because it provides a brief, standardized assessment of body image-related thoughts, emotions, and behaviors. Although the instrument was originally developed for individuals with cancer, its items assess general aspects of body image that are applicable beyond clinical settings. In the present study, the scale was considered appropriate for evaluating body image perceptions among healthy adults, although its original development in a clinical population should be considered when interpreting the findings.

### Scoring system

For the Self-Confidence Scale, item responses were scored from 1 to 5, yielding a total possible score range of 48–240, where higher scores indicated greater self-confidence (18). For the Body Image Scale, total scores were calculated by summing responses across all items, with possible scores ranging from 10 to 50. All Body Image Scale items were reverse-scored so that higher scores represented a more positive body image perception, lower body image dissatisfaction, and reduced appearance-related distress (20).

### Validity and reliability

The Self-Confidence Scale and the Body Image Scale were administered in their Arabic versions. Where necessary, the instruments were translated using a forward–backward translation procedure to ensure semantic and conceptual equivalence, and the translated versions were reviewed by a panel of 13 experts for clarity, cultural relevance, and content validity. A pilot study was conducted to confirm the comprehensibility of the questionnaires before data collection. Reliability was assessed using the test–retest method and internal consistency analysis. Cronbach's alpha coefficient was 0.83 for the Self-Confidence Scale and 0.81 for the Body Image Scale, indicating good internal consistency in the study population.

### Pilot study

A pilot study was conducted on 30 participants (15 bodybuilders and 15 non-exercising adults) to assess the feasibility of the study procedures, clarity of questionnaire items, and reliability of the instruments. The pilot study confirmed that the questionnaire was understandable and appropriate for the target population, and no substantial modifications to the validated instruments were required. Minor administrative adjustments were made regarding the explanation of instructions and data collection procedures. The pilot participants were excluded from the final sample and were not included in the statistical analysis.

### Data analysis

Statistical analysis was performed using the Statistical Package for the Social Sciences (SPSS) version 25. Descriptive statistics were used to summarize the data, where categorical variables were presented as frequencies and percentages, while continuous variables were expressed as means and standard deviations (Mean ± SD). Comparisons between the case and control groups for categorical variables were conducted using the chi-square test, whereas independent samples *t*-tests were applied to compare mean scores of self-confidence and body image scale items as well as total scale scores between the two groups. Effect sizes were calculated using Cohen's d to estimate the magnitude of differences between the case and control groups. Cohen's d values were interpreted as small (0.20–0.49), moderate (0.50–0.79), and large (≥0.80) effects. Effect sizes were reported alongside *p-*values to provide a more comprehensive interpretation of the findings. Binary logistic regression analysis was subsequently performed to identify independent predictors of group membership using total self-confidence and total body image scale scores as explanatory variables. Regression coefficients (B), standard errors (S.E.), Wald statistics, odds ratios [Exp(B)], and 95% confidence intervals (95% CI) were reported. A binary logistic regression analysis was performed with bodybuilding status (case vs. control) as the dependent variable and total self-confidence and body image scores as the independent variables to examine their independent association with membership in the bodybuilding group. The model was used to identify factors associated with group membership and was not intended to establish causal relationships. A *p-*value of less than 0.05 was considered statistically significant throughout the analysis.

### Ethical approval

The study protocol was approved by the Ethics Committee of Al-Qadisiyah University (Ethics Committee No.: 2025/201) and was conducted in accordance with the ethical principles outlined in the Declaration of Helsinki. Written informed consent was obtained from all participants before data collection. Participants were informed about the purpose of the study, and the confidentiality of the collected information was maintained throughout the research process.

## Results

Table 1 summarizes the sociodemographic characteristics of participants in the case and control groups. The majority of participants were aged 18–24 years (48.9%), reported sufficient monthly income (74.5%), had undergraduate education (39.4%), and were married (69.1%). No statistically significant differences were observed between the case and control groups in terms of mean age, age categories, monthly income, educational level, or marital status (all *p* > 0.05).

**Table 1:** Sociodemographic characteristics of participants in the case and control groups.

Variables	Case *n* = 94 (50.0%)	Control *n* = 94 (50.0%)	Total *n* = 188 (100%)	*P* value
Age (years)				
Mean ± SD	25.81 ± 5.5	26.99 ± 5.8	26.40 ± 5.7	0.157
Age categories				
18–24 years	48 (52.2)	44 (47.8)	92 (48.9)	0.237
25–30 years	24 (57.1)	18 (42.9)	42 (22.3)	
31–38 years	22 (40.7)	32 (59.3)	54 (28.8)	
Monthly income				
Sufficient	72 (51.4)	68 (48.6)	140 (74.5)	0.308
Insufficient	22 (45.8)	26 (54.2)	48 (25.5)	
Educational level				
Primary	22 (66.7)	11 (33.3)	33 (17.6)	0.163
Secondary	36 (50.0)	36 (50.0)	72 (38.3)	
Undergraduate	32 (43.2)	42 (56.8)	74 (39.4)	
Postgraduate	4.0 (44.4)	5.0 (55.6)	9.0 (4.7)	
Marital status				
Single	28 (48.3)	30 (51.7)	58 (30.9)	0.437
Married	66 (50.8)	64 (49.2)	130 (69.1)	

Categorical variables were presented as frequencies and percentages, while continuous variables were summarized using means and standard deviations. Group comparisons were performed using the chi-square test and independent samples *t*-test.

Table 2 presents the comparison of self-confidence item scores between the case and control groups using independent samples *t*-tests. Several items showed statistically significant differences between groups. Participants in the case group had higher mean scores than the control group for items related to social interaction, including liking to mix with people (*p* = 0.001) and meeting new people as an enjoyable experience (*p* = 0.001). Significant differences were also observed for items related to self-evaluation and appearance concerns, including feeling upset with oneself (*p* = 0.001), dissatisfaction with physical attractiveness (*p* = 0.001), and increased self-criticism (*p* = 0.001). Additionally, significant differences were found in several items related to optimism, communication confidence, academic challenge-seeking, and interpersonal confidence (all *p* < 0.05). In contrast, no statistically significant differences were observed between groups for the majority of self-confidence items related to academic confidence, public speaking, social comfort, and interpersonal relationships (all *p* > 0.05). The total self-confidence score was significantly higher in the case group compared with the control group (148.37 ± 25.6 vs. 137.32 ± 26.7, *p* = 0.004), with a small effect size (Cohen's d = 0.42).

**Table 2:** Comparison of self-confidence scores between case and control groups.

Variables	Case *n* = 94 (50.0%)	Control *n* = 94 (50.0%)	*t*-value	df	*P* value
**1. I like mixing with people**					
Mean ± SD	3.00 ± 1.2	2.32 ± 0.8	4.36	168	0.001
**2. I felt very upset with myself in the past period**					
Mean ± SD	3.02 ± 1.2	2.18 ± 0.8	5.50	169	0.001
**3. It bothers me that I am not physically attractive**					
Mean ± SD	3.04 ± 1.1	1.49 ± 0.5	11.5	130	0.001
**4. I find having a satisfying romantic relationship with someone of the opposite sex enjoyable**					
Mean ± SD	2.91 ± 1.1	3.03 ± 1.1	−0.694	185	0.488
**5. I am happier now compared to a few weeks ago**					
Mean ± SD	3.13 ± 1.2	2.98 ± 1.2	0.850	185	0.396
**6. I am satisfied and happy with my physical appearance**					
Mean ± SD	2.96 ± 1.2	3.07 ± 1.1	−0.683	184	0.495
**7. I often feel shy when speaking in front of a group of people**					
Mean ± SD	2.85 ± 1.1	2.87 ± 1.0	−0.132	185	0.895
**8. Although I would like to meet more people, I dislike going out and meeting them as it wastes my time**					
Mean ± SD	3.00 ± 1.2	3.03 ± 1.1	−0.186	185	0.853
**9. Academic performance is a field where I can show my competence and abilities and gain recognition for my achievements**					
Mean ± SD	2.89 ± 1.1	2.98 ± 1.2	−0.502	185	0.616
**10. I look more attractive than the average person**					
Mean ± SD	3.09 ± 1.1	2.90 ± 1.0	1.08	184	0.280
**11. I am afraid of thinking about speaking in front of an audience**					
Mean ± SD	2.85 ± 1.1	3.04 ± 1.1	−1.14	185	0.253
**12. I often feel hesitant even in situations I have handled successfully before**					
Mean ± SD	2.89 ± 1.1	2.90 ± 1.1	−0.064	185	0.949
**13. My confidence in my mental ability to achieve my academic and professional goals is low**					
Mean ± SD	3.06 ± 1.1	3.13 ± 1.1	−0.376	185	0.707
**14. I often feel less competent than most people around me in dealing with others**					
Mean ± SD	3.00 ± 1.1	2.90 ± 1.0	0.573	184	0.567
**15. When I must speak in front of a group, I usually feel I can express myself clearly and effectively**					
Mean ± SD	3.02 ± 1.1	2.87 ± 1.0	0.906	184	0.366
**16. I am fortunate to be as physically attractive as I am**					
Mean ± SD	2.89 ± 1.1	3.03 ± 1.1	−0.831	186	0.407
**17. I lack some important abilities needed for academic success**					
Mean ± SD	3.06 ± 1.1	2.98 ± 1.2	0.494	185	0.622
**18. I admit that I am not as good a student as many of my peers**					
Mean ± SD	3.00 ± 1.1	3.07 ± 1.1	−0.442	185	0.659
**19. Meeting new people is an enjoyable experience that I always look forward to**					
Mean ± SD	3.02 ± 1.1	2.32 ± 0.8	4.61	172	0.001
**20. I have been more self–critical in recent days than usual**					
Mean ± SD	2.91 ± 1.1	1.76 ± 0.5	9.20	129	0.001
**21. I always feel comfortable and happy at parties or social gatherings**					
Mean ± SD	2.91 ± 1.1	2.91 ± 1.1	0.00	186	1.00
**22. My doubts about my academic abilities are less than those of most of my peers**					
Mean ± SD	3.13 ± 1.2	2.97 ± 1.2	0.909	186	0.364
**23. I face more problems than others in forming romantic relationships with the opposite sex**					
Mean ± SD	2.87 ± 1.1	3.00 ± 1.1	−0.754	185	0.452
**24. My confidence in speaking clearly in front of people is decreasing more now than ever before**					
Mean ± SD	3.06 ± 1.1	2.90 ± 1.0	0.993	185	0.322
**25. It bothers me that I am not at the same intellectual level as others**					
Mean ± SD	2.91 ± 1.1	3.02 ± 1.1	0.920	185	0.536
**26. When things go wrong, I am usually confident that I can handle them successfully**					
Mean ± SD	3.13 ± 1.2	2.97 ± 1.2	0.839	186	0.364
**27. I am more concerned than others about my ability to build successful social relationships**					
Mean ± SD	2.87 ± 1.1	2.79 ± 1.0	0.124	181	0.597
**28. My self-confidence is higher than many people I know**					
Mean ± SD	3.06 ± 1.1	3.05 ± 1.2	0.342	184	0.950
**29. I feel fear and anxiety when thinking about dating**					
Mean ± SD	2.91 ± 1.1	2.79 ± 1.0	0.513	184	0.437
**30. Many people consider my physical appearance unattractive**					
Mean ± SD	3.13 ± 1.2	3.14 ± 1.2	0.974	185	0.952
**31. When studying a new subject, I am confident I will excel among the top students**					
Mean ± SD	2.87 ± 1.1	2.79 ± 1.0	0.124	181	0.597
**32. I am less capable than most people in speaking in front of a group**					
Mean ± SD	3.06 ± 1.1	2.84 ± 1.0	0.807	185	0.159
**33. When attending social gatherings, I often feel nervous and tired**					
Mean ± SD	2.91 ± 1.0	2.96 ± 1.1	0.724	185	0.794
**34. I sometimes avoid activities that require being in a group**					
Mean ± SD	3.17 ± 1.2	2.95 ± 1.1	0.427	185	0.205
**35. When I have exams or assignments, I am confident I will succeed**					
Mean ± SD	2.91 ± 1.0	3.00 ± 1.1	0.843	185	0.598
**36. When meeting new people, I communicate better than many others**					
Mean ± SD	3.17 ± 1.2	2.84 ± 1.0	0.056	182	0.049
**37. I feel more decisive and confident now than at any other time**					
Mean ± SD	2.91 ± 1.0	2.97 ± 1.1	0.665	185	0.745
**38. I sometimes avoid someone of the opposite sex with whom I might form a romantic relationship due to tension and anxiety**					
Mean ± SD	3.19 ± 1.1	2.95 ± 1.2	0.853	185	0.165
**39. I wish I could change my physical appearance**					
Mean ± SD	3.02 ± 1.1	3.05 ± 1.1	0.853	185	0.847
**40. My anxiety about speaking in front of an audience has significantly decreased compared to others**					
Mean ± SD	3.87 ± 1.2	2.84 ± 1.0	6.214	182	0.001
**41. I feel more optimistic and positive than at any previous time**					
Mean ± SD	3.31 ± 1.2	2.86 ± 1.1	2.491	183	0.014
**42. Finding a suitable partner from the opposite sex for a romantic relationship is a problem for me**					
Mean ± SD	3.64 ± 1.1	3.10 ± 1.1	3.249	185	0.001
**43. If I were more confident when speaking or discussing matters, my life would be better**					
Mean ± SD	3.31 ± 1.2	2.90 ± 1.1	2.274	183	0.024
**44. I always seek challenging academic activities because I am confident I can perform better than others**					
Mean ± SD	3.61 ± 1.1	2.99 ± 1.0	3.792	184	0.001
**45. I can easily obtain many dating opportunities**					
Mean ± SD	3.30 ± 1.3	2.86 ± 1.1	2.425	183	0.016
**46. I feel less comfortable in groups compared to other members**					
Mean ± SD	3.63 ± 1.1	3.10 ± 1.1	3.157	185	0.002
**47. My confidence in dealing with the opposite sex has increased recently**					
Mean ± SD	3.31 ± 1.2	2.90 ± 1.1	2.082	185	0.039
**48. If my physical appearance were better, I would be more attractive to the opposite sexs**					
Mean ± SD	3.55 ± 1.2	2.99 ± 1.0	3.319	180	0.001
**Total self-confidence scores**	148.37 ± 25.6	137.32 ± 26.7	2.891	Cohen's d: 0.42	0.004

Continuous variables were summarized using means and standard deviations. Group comparisons were performed using independent samples *t*-test.

Table 3 presents the comparison of body image scale scores between the case and control groups. Significant differences were observed between groups for several body image items. Compared with the control group, participants in the case group had different scores for dissatisfaction with appearance when undressed (*p* = 0.004), difficulty looking at their body while naked (*p* = 0.003), avoidance of people because of appearance concerns (*p* = 0.003), and feelings of reduced physical attractiveness due to illness or treatment (*p* = 0.029). No statistically significant differences were observed between the two groups for items related to embarrassment or discomfort about physical appearance, dissatisfaction with appearance when dressed, reduced femininity/masculinity, reduced sexual attractiveness, feelings of body incompleteness, or dissatisfaction with scars (all *p* > 0.05). The total body image scale score differed significantly between groups, with higher scores observed in the case group compared with the control group (32.69 ± 7.6 vs. 29.70 ± 5.5, *p* = 0.002, Cohen's d = 0.45).

**Table 3:** Comparison of body image scale scores among participants in the case and control groups.

Variables	Case *n* = 94 (50.0%)	Control *n* = 94 (50.0%)	*t*-value	df	*P* value
**1. Have you felt embarrassed or uncomfortable about your physical appearance?**					
Mean ± SD	3.33 ± 1.2	3.10 ± 1.1	1.334	185	0.184
**2. Have you felt less physically attractive due to illness or treatment?**					
Mean ± SD	3.29 ± 1.2	2.90 ± 1.1	2.206	185	0.029
**3. Have you been dissatisfied with your appearance when dressed?**					
Mean ± SD	3.24 ± 1.2	2.99 ± 1.0	1.540	185	0.125
**4. Have you been dissatisfied with your appearance when undressed?**					
Mean ± SD	3.28 ± 1.2	2.78 ± 1.0	2.938	179	0.004
**5. Have you felt less feminine/masculine due to illness or treatment?**					
Mean ± SD	3.20 ± 1.2	3.14 ± 1.1	0.368	182	0.713
**6. Have you found it difficult to look at your body while naked?**					
Mean ± SD	3.29 ± 1.2	2.78 ± 1.0	3.059	181	0.003
**7. Have you felt less sexually attractive due to illness or treatment?**					
Mean ± SD	3.26 ± 1.2	3.14 ± 1.1	0.662	184	0.509
**8. Have you avoided people because of your feelings about your appearance?**					
Mean ± SD	3.45 ± 1.2	2.94 ± 1.0	3.027	181	0.003
**9. Have you felt that your body is less complete due to illness or treatment?**					
Mean ± SD	3.18 ± 1.3	3.05 ± 1.1	0.712	181	0.477
**10 Have you felt dissatisfied with your scars?**					
Mean ± SD	3.18 ± 1.2	2.89 ± 1.1	1.638	183	0.103
**Total body image scale scores**	32.69 ± 7.6	29.70 ± 5.5	3.150	Cohen's d: 0.45	0.002

Continuous variables were summarized using means and standard deviations. Group comparisons were performed using independent samples *t*-test. All body image scale items were reverse-scored; therefore, higher scores indicate more positive body image perception and lower body image distress.

Binary logistic regression analysis was performed to examine the association of total self-confidence and body image scores with study group membership (case vs. control). Total self-confidence scores were significantly associated with group membership (B = −0.012, *p* = 0.040), with an odds ratio of 0.988 (95% CI: 0.976–0.999). Similarly, total body image scale scores showed a statistically significant association with group membership (B = −0.065, *p* = 0.009), with an odds ratio of 0.937 (95% CI: 0.892–0.984). The regression coefficients, odds ratios, and confidence intervals are presented in Table 4.

**Table 4:** Logistic regression model identifying determinants of group membership using total self-confidence and body image scale scores.

Variables	B	S.E.	Wald	df	Sig.	Exp (B)	95% C.I. for EXP(B)	
							Lower	Upper
Total self-confidence scores	−0.012	0.006	4.234	1	0.040	0.988	0.976	0.999
Total body image scale scores	−0.065	0.025	6.790	1	0.009	0.937	0.892	0.984

## Discussion

The present case-control study investigated differences in self-confidence and body image between adults engaged in daily bodybuilding exercise and non-exercising adults in Al-Diwaniyah City, Iraq. The findings demonstrated that participants involved in regular bodybuilding exercise had significantly higher total self-confidence scores and more positive body image perceptions compared with the control group. These findings support the hypothesis that regular participation in bodybuilding is associated with higher levels of self-confidence and more positive body image. However, the study design does not allow conclusions regarding whether bodybuilding leads to these outcomes or whether individuals with these characteristics are more likely to participate in bodybuilding. Although statistically significant differences were observed between the bodybuilding and control groups, the calculated effect sizes were small (Cohen's d = 0.42 for self-confidence and 0.45 for body image). These findings indicate that the magnitude of the observed differences was modest despite their statistical significance. Therefore, the results should be interpreted as demonstrating statistically significant but relatively small differences between groups. Furthermore, because of the observational case-control design, the findings indicate associations rather than causal relationships, and longitudinal or interventional studies are needed to clarify the direction of these associations.

The present study revealed significantly higher total self-confidence scores among participants in the bodybuilding group compared with non-exercising adults. This finding is consistent with previous studies reporting that regular physical activity and resistance training are associated with enhanced self-confidence, psychological resilience, and social confidence (21, 22). Exercise participation may improve self-confidence through several mechanisms, including enhanced physical competence, achievement of personal fitness goals, improved appearance satisfaction, and greater social interaction within fitness environments (21). Individuals who regularly engage in bodybuilding exercise may also develop stronger perceptions of self-discipline, competence, and personal control, which contribute positively to overall self-confidence (22).

Interestingly, the item-level analysis demonstrated a complex pattern of self-confidence among bodybuilders. Participants in the case group scored significantly higher in several positive domains such as optimism, communication confidence, social interaction, and willingness to engage in academic challenges. However, they also reported higher scores on certain items related to self-criticism, emotional sensitivity, and concerns about physical attractiveness. These findings suggest that bodybuilding exercise may simultaneously enhance general self-confidence while increasing awareness and evaluation of physical appearance. Similar observations were reported by Dobrich (23), who suggested that individuals engaged in bodybuilding may experience increased appearance-related concerns despite improved self-perceived attractiveness. The author interpret this finding as evidence that bodybuilding culture may encourage continuous self-monitoring and pursuit of physical perfection, resulting in heightened sensitivity toward body image and self-evaluation (23). Although participants engaged in bodybuilding demonstrated higher overall self-confidence scores, the item-level findings revealed a more complex psychological pattern. Bodybuilders also reported higher levels of self-criticism and greater concerns related to physical attractiveness, suggesting that increased confidence may coexist with heightened body monitoring and appearance-related evaluation. This finding may reflect the influence of bodybuilding culture, where continuous comparison, pursuit of muscular improvement, and emphasis on physical appearance can contribute to perfectionistic tendencies and increased sensitivity toward perceived physical shortcomings. Some individuals involved in bodybuilding may experience a dual effect, with improvements in physical self-perception and confidence accompanied by increased appearance concerns and risk of maladaptive body image attitudes, including features related to muscle dysmorphia (22, 23). Therefore, the psychological impact of bodybuilding should not be interpreted as uniformly positive; rather, it may involve both beneficial effects on competence, social confidence, and self-perception and potential challenges related to excessive appearance focus.

The present study also found significantly more positive body image perception among bodybuilders compared with controls. Participants engaged in regular bodybuilding exercise reported lower dissatisfaction with their appearance, reduced discomfort regarding body exposure, and less avoidance of social situations because of appearance concerns. These findings are consistent with previous research demonstrating that physical activity and resistance training contribute positively to body image satisfaction and appearance-related confidence (24). Improvements in muscular strength, body composition, and perceived physical attractiveness may explain the more favorable body image observed among participants in the case group (25).

Nevertheless, not all body image domains showed statistically significant differences between groups. Items related to embarrassment about appearance, femininity/masculinity, sexual attractiveness, and body incompleteness were relatively similar between the two groups. This finding may indicate that some aspects of body image are influenced not only by physical exercise but also by broader psychological, cultural, and social factors (26). In Middle Eastern societies, body image perceptions may be shaped by cultural expectations, gender norms, and social comparisons, which may persist regardless of exercise participation (27). The bodybuilding exercise may improve appearance satisfaction primarily through visible physical changes, while deeper emotional and sociocultural perceptions of body image may remain relatively stable (28). Although the Body Image Scale has demonstrated good psychometric properties and was considered suitable for assessing general body image perceptions in the present study, it was originally developed for individuals with cancer. Consequently, some items addressing appearance-related distress associated with illness or treatment may be less directly applicable to a healthy bodybuilding population. Although all participants completed the same instrument under identical conditions and the scale demonstrated good internal consistency in the current sample, the findings should be interpreted with caution. Future studies should consider using body image instruments specifically developed and validated for healthy adults or physically active populations to provide a more comprehensive assessment of body image in bodybuilding settings.

The logistic regression analysis further demonstrated that higher self-confidence and more positive body image were independently associated with membership in the bodybuilding group. However, the observed odds ratios were close to 1, indicating relatively small effect sizes. These findings should therefore be interpreted as demonstrating statistical associations rather than strong predictive relationships. Because of the observational case-control design, the temporal direction of these associations cannot be determined, and it remains possible that individuals with higher self-confidence and more positive body image are more likely to participate in bodybuilding rather than bodybuilding leading to these characteristics. Individuals with higher self-confidence may be more motivated to participate in structured physical exercise, while improvements in physical appearance and performance may subsequently reinforce positive self-perception. This reciprocal relationship between exercise, self-confidence, and body image has been highlighted in previous psychological and behavioral research (29). The findings of the current study may also be interpreted within the framework of social and behavioral psychology (30). Participation in bodybuilding centers provides opportunities for social interaction, peer support, and achievement-oriented environments that may strengthen self-confidence and social competence. Furthermore, regular exercise may reduce stress and improve emotional wellbeing through neurobiological mechanisms such as endorphin release and improved mood regulation. Therefore, the observed psychological benefits among bodybuilders are likely multifactorial and influenced by both physiological and psychosocial pathways. Despite these positive findings, the present results should be interpreted cautiously. Although bodybuilders demonstrated higher overall self-confidence and more positive body image, some appearance-related concerns and self-critical tendencies remained elevated. This finding highlights the importance of promoting balanced and healthy exercise behaviors while preventing excessive preoccupation with physical appearance or unrealistic body ideals. Additionally, the observed associations may have been influenced by unmeasured confounding factors, including baseline psychological characteristics, personality traits, socioeconomic status, dietary habits, social support, and previous participation in sports or physical activity. As these variables were not assessed or controlled for, they may have partly explained the differences in self-confidence and body image observed between the bodybuilding and non-exercising groups.

### Strengths and limitations

The present study has several strengths. First, it utilized a case-control design that enabled direct comparison between adults engaged in regular bodybuilding exercise and non-exercising adults. Second, validated instruments were used to assess self-confidence and body image, which enhanced the reliability of the measurements. Third, the inclusion of participants from multiple bodybuilding and fitness centers improved the diversity of the bodybuilding group. In addition, frequency matching based on key sociodemographic characteristics helped reduce potential differences between the study groups.

However, several limitations should be acknowledged. The use of purposive non-probability sampling may restrict the generalizability of the findings, as individuals who participate in bodybuilding programs may differ from non-exercising adults in terms of motivation, personality characteristics, lifestyle behaviors, and baseline psychological attributes, potentially introducing selection bias. Moreover, recruiting cases and controls from different settings (fitness centers vs. university-based populations) may have resulted in additional selection effects despite efforts to achieve comparability through matching. Although matching was performed for selected sociodemographic characteristics, residual confounding remains possible due to unmeasured factors, including gender, previous sports participation, psychological status, dietary behaviors, social support, socioeconomic conditions, exercise motivation, and other lifestyle factors. Another important limitation is that participant gender was not recorded and therefore could not be considered during matching or statistical analysis. Gender is known to influence body image, self-confidence, exercise motivation, and appearance-related concerns. Consequently, it was not possible to determine whether the observed associations differed between men and women or whether gender may have confounded the findings. Future studies should collect and analyze gender-specific data to improve the interpretation and generalizability of the results. In addition, detailed bodybuilding-related characteristics, including training duration beyond the minimum eligibility criterion, weekly training frequency, session intensity, training volume, years of experience, and competitive vs.recreational participation, were not collected. These factors may substantially influence psychological outcomes, including self-confidence, body image, and appearance-related concerns. Consequently, it was not possible to examine dose-response relationships or determine whether the observed associations differed according to the level or type of bodybuilding participation. Recreational exercisers and experienced or competitive bodybuilders may have distinct psychological profiles, and the absence of these data limits the interpretation and generalizability of the findings. Future studies should collect detailed training characteristics to better characterize participants and explore their potential influence on psychological outcomes. The use of self-reported questionnaires may have introduced recall and social desirability biases, particularly for subjective measures related to body image and self-confidence. The sample size was determined based on feasibility rather than formal power estimation, which may have influenced statistical precision. Furthermore, multiple item-level comparisons were conducted without adjustment for multiple testing, increasing the possibility of Type I error; therefore, these findings should be interpreted cautiously. Moreover, the study was conducted in a single city, which may limit the applicability of the results to other populations and cultural contexts. Furthermore, although the Body Image Scale demonstrated acceptable reliability in the present study, it was originally designed for individuals with cancer rather than healthy adults. Some items referring to illness- or treatment-related changes may have limited relevance for bodybuilders and may not fully capture body image concerns specific to resistance training, muscularity, or physique development. Therefore, caution is warranted when interpreting these findings, and future research should employ instruments specifically validated for healthy or athletic populations. The logistic regression findings should be interpreted cautiously, as the model examined associations with group membership rather than causal relationships. The observed relationships between self-confidence, body image, and bodybuilding status are descriptive, and the small effect sizes indicated by odds ratios close to 1 suggest limited predictive value. Longitudinal studies are needed to clarify the direction and causality of these associations. Although statistically significant differences were identified, the observed effect sizes were small, suggesting that the practical magnitude of the differences between bodybuilders and non-exercising adults was modest.

## Conclusion

The present study demonstrated that adults engaged in daily bodybuilding exercise exhibited significantly higher self-confidence and more positive body image perception compared with non-exercising adults. Adults who regularly participated in bodybuilding exhibited higher self-confidence and more positive body image than non-exercising adults. These findings indicate an association between bodybuilding participation and psychological outcomes but do not establish causality. Individuals with greater self-confidence and a more positive body image may be more likely to engage in bodybuilding, or bodybuilding participation may be associated with these characteristics. Prospective longitudinal or intervention studies are needed to determine the direction of these relationships. However, some bodybuilders also demonstrated increased emotional sensitivity and appearance-related self-evaluation, suggesting that bodybuilding exercise may have complex psychological effects. The findings highlight the potential role of regular physical exercise in promoting mental health and positive self-perception among adults. Healthcare professionals, psychologists, fitness trainers, and public health specialists should encourage balanced participation in physical exercise programs while also promoting healthy body image attitudes and realistic appearance expectations. Future longitudinal and multicenter studies are recommended to further explore the causal relationship between bodybuilding exercise, self-confidence, and body image across different cultural settings and age groups.

## Data Availability

The raw data supporting the conclusions of this article will be made available by the authors, without undue reservation.
